# High Specificity in Circulating Tumor Cell Identification Is Required for Accurate Evaluation of Programmed Death-Ligand 1

**DOI:** 10.1371/journal.pone.0159397

**Published:** 2016-07-26

**Authors:** Jennifer L. Schehr, Zachery D. Schultz, Jay W. Warrick, David J. Guckenberger, Hannah M. Pezzi, Jamie M. Sperger, Erika Heninger, Anwaar Saeed, Ticiana Leal, Kara Mattox, Anne M. Traynor, Toby C. Campbell, Scott M. Berry, David J. Beebe, Joshua M. Lang

**Affiliations:** 1 Carbone Cancer Center, University of Wisconsin-Madison, Madison, Wisconsin, United States of America; 2 Department of Biomedical Engineering, University of Wisconsin-Madison, Madison, Wisconsin, United States of America; 3 Department of Medicine, University of Wisconsin-Madison, Madison, Wisconsin, United States of America; The Ohio State University, UNITED STATES

## Abstract

**Background:**

Expression of programmed-death ligand 1 (PD-L1) in non-small cell lung cancer (NSCLC) is typically evaluated through invasive biopsies; however, recent advances in the identification of circulating tumor cells (CTCs) may be a less invasive method to assay tumor cells for these purposes. These liquid biopsies rely on accurate identification of CTCs from the diverse populations in the blood, where some tumor cells share characteristics with normal blood cells. While many blood cells can be excluded by their high expression of CD45, neutrophils and other immature myeloid subsets have low to absent expression of CD45 and also express PD-L1. Furthermore, cytokeratin is typically used to identify CTCs, but neutrophils may stain non-specifically for intracellular antibodies, including cytokeratin, thus preventing accurate evaluation of PD-L1 expression on tumor cells. This holds even greater significance when evaluating PD-L1 in epithelial cell adhesion molecule (EpCAM) positive and EpCAM negative CTCs (as in epithelial-mesenchymal transition (EMT)).

**Methods:**

To evaluate the impact of CTC misidentification on PD-L1 evaluation, we utilized CD11b to identify myeloid cells. CTCs were isolated from patients with metastatic NSCLC using EpCAM, MUC1 or Vimentin capture antibodies and exclusion-based sample preparation (ESP) technology.

**Results:**

Large populations of CD11b^+^CD45^lo^ cells were identified in buffy coats and stained non-specifically for intracellular antibodies including cytokeratin. The amount of CD11b^+^ cells misidentified as CTCs varied among patients; accounting for 33–100% of traditionally identified CTCs. Cells captured with vimentin had a higher frequency of CD11b^+^ cells at 41%, compared to 20% and 18% with MUC1 or EpCAM, respectively. Cells misidentified as CTCs ultimately skewed PD-L1 expression to varying degrees across patient samples.

**Conclusions:**

Interfering myeloid populations can be differentiated from true CTCs with additional staining criteria, thus improving the specificity of CTC identification and the accuracy of biomarker evaluation.

## Introduction

Circulating tumor cells (CTCs) are rare cells that can be detected in the blood of patients with solid tumors and have been explored as a form of liquid biopsy [1–3]. Enumeration of CTCs following epithelial cell adhesion molecule (EpCAM) based capture serves as a prognostic and predictive biomarker in different malignancies such as prostate [4] and breast cancer [5], as well as non-small cell lung cancer (NSCLC) [6]. However, the limited biological readout of enumeration does not inform on the wide range of therapeutic targets for patients with advanced cancer. Given the variety of new treatments in early phase and advanced clinical trials, there is a critical need to develop new biomarkers that may predict the benefits of these treatments and identify early signs of therapeutic resistance.

Lung cancer is one of the most common and lethal types of cancer, with a 5-year survival rate of ~15% when diagnosed with metastatic disease [7]. Immunotherapy has revolutionized the treatment of advanced lung cancer, leading to durable responses and improved survival benefit in a subset of patients with NSCLC [8–10]. Therefore, recent therapeutic advances in checkpoint inhibition have given rise to interest in developing predictive biomarkers. Programmed death ligand-1 (PD-L1) expression is quantified on tumor biopsies prior to PD-L1 based treatment of patients with NSCLC, yet the invasive nature of biopsy often leads clinicians to test archived biopsies that may not be representative of the disease after exposure to chemotherapy or targeted therapies. Thus, evaluating CTCs for PD-L1 expression could be useful as a surrogate to tumor biopsies.

The standard molecular definition of a CTC is based on positive detection of cells with an intact nucleus that express cytokeratin (CK) but are negative for CD45, a white blood cell (WBC) marker [11]. While FDA cleared, this limited definition of a CTC does not account for the diversity of WBCs in circulation with low or absent expression of CD45 such as neutrophils [12], myeloid-derived suppressor cells (MDSCs) [13], or other immature blasting myeloid populations [14]. Confounding the issue to an even greater extent is the increase in neutrophil number in patients with progressive cancer [15], as well as evidence that neutrophils stain positive for CK [16], raising concerns over the specificity of traditional CTC identification approaches.

These factors may be even more significant when evaluating CTC biomarkers amidst phenomena such as epithelial-mesenchymal transition (EMT) given the expression of EMT-related proteins such as Vimentin [17] and CD44 [18] on neutrophils. However, EMT-based CTC capture may be required in NSCLC as traditional capture with EpCAM was shown to be effective in only 20% of samples from patients with metastatic NSCLC [19], as opposed to 57% from those with prostate cancer [11]. Furthermore, PD-L1 can be expressed to varying degrees by neutrophils [20] and myeloid subsets [21], complicating blood sample analysis, and confounding any assay purporting to evaluate CTC immune biomarkers. These issues are particularly challenging for antibody-independent methods of CTC capture (i.e., filter based methods or depletion methodologies), as size based criteria or nuclear morphology would not exclude many of these WBC subsets given their complex nuclear structures, high granularity and large size [14]. Depletion of myeloid subsets prior to antibody based enrichment has been employed by others [22, 23], but the efficacy of these approaches is unclear as stains for depleted populations were not ultimately included during CTC identification. Regardless of the technique used to isolate CTCs, these potentially interfering WBC populations must be excluded during CTC identification to accurately evaluate CTC biomarkers.

In the current study, we quantify the impact of contaminating WBCs on the analysis of PD-L1 in putative CTCs. Interfering WBC populations are excluded by staining for CD11b, which is strongly expressed on CD45^lo^ myeloid populations, including both MDSCs and neutrophils [12, 24, 25]. CTCs are captured with exclusion-based sample preparation (ESP) technology; which employs magnetic force and surface tension for gentle and efficient antibody-based cell capture. Two versions of ESP technology are used: either the manual Versatile Exclusion-based Rare Sample Analysis (VERSA) device [1], or the automated version using the Sliding Lid for Immobilized Droplet Extraction (SLIDE) technology [2]. We further employ image analysis software (JEX) [26] to objectively identify CTCs and quantify PD-L1 biomarker expression. We observe a population of CD11b^+^CD45^lo^ cells which are misidentified as CTCs based on CK staining, and which ultimately interfere with evaluation of biomarkers such as PD-L1. By increasing the accuracy of CTC identification, biomarker evaluation for checkpoint inhibitors becomes more specific across a cohort of patients with NSCLC and has greater potential to accurately predict therapeutic response.

## Methods

### Patient Samples

Peripheral blood was collected from 19 patients with metastatic NSCLC (S1 Table) with informed written consent under University of Wisconsin IRB approved protocol. A maximum of 50 mL of blood was obtained at any given blood draw using EDTA vacutainers (BD). Whole blood was diluted 1:1 with Hank’s balanced salt solution (HBSS, Lonza) and 30 mL of diluted blood was underlaid with 10 mL of ficoll-paque PLUS (GE Healthcare) per 50 mL conical tube. The blood was centrifuged for 40 min at 974 g, and resulting buffy coats were washed once with HBSS. Peripheral blood mononuclear cells (PBMCs) were incubated with commercially available, pre-conjugated, anti-CD45 magnetic beads (Miltenyi) in a buffer containing 2 mM EDTA (Fisher Scientific) and 0.5% bovine serum albumin (BSA, Sigma-Aldrich) in phosphate buffered saline (PBS, Hyclone), after which CD45^+^ cells were removed using magnetic LS MACS columns (Miltenyi).

### Paramagnetic Particle (PMP) Preparation

Streptavidin coupled PMPs (Dynabeads FlowComp Flexi, Invitrogen) at a concentration of 250 μg per reaction were used for all experiments. The PMPs were washed three times and resuspended in 0.1% Tween-20 (Fisher Scientific) in PBS prior to use. PMP solutions were then incubated under agitation with 0.5 μg of a single capture antibody, against either EpCAM (R&D Systems, clone TROP1), Mucin 1 (MUC1, Biolegend, clone 16A), or the extracellular region of Vimentin (Vim, Tonbo, clone V9) [27], biotinylated according to the manufacturer’s recommendations. Prior to use, bead conjugates were washed an additional three times and held in 0.1% BSA in PBS.

### CTC Isolation

CTCs were captured from CD45 depleted PBMCs with antibody labelled PMPs. CD45 depleted PBMCs were incubated on a rotator with antibody-labelled PMPs in a buffer containing 0.01% Tween-20, 2 mM EDTA, and 0.1% BSA in PBS for 30 min at 5°C. Cells bound to PMPs were isolated with ESP technology: using either a manual (VERSA) [1], or automated (SLIDE) [2] device. PMP-bound cells were moved through staining and washing wells prior to imaging at 10x or 40x with a Nikon Eclipse Ti fluorescent microscope (Nikon) and NIS-Elements AR 4.10 software (Nikon) either directly in VERSA devices or after a final transfer to glass-bottom chamber slides.

### Fluorescent Staining

For all staining, cells were blocked with 0.1% BSA prior to extracellular staining, then, if intracellularly stained, cells were treated with Fixation and Permeabilization Solution (BD) and stained for intracellular markers in Perm/Wash Buffer (BD). Flow cytometry was performed on an LSR II and analyzed in FlowJo software, both courtesy of the University of Wisconsin Flow Core facility. Stains: Hoechst, and antibodies against PD-L1 (BD, MIH1), MUC1 (Biolegend, 16A), CD11b (Biolegend M1/70), CD34 (Biolegend, 581), CD45 (Biolegend, H130; Tonbo, HI30; or abcam GA90), pan-Cytokeratin (pCK, abcam, C-11), and isotype control, IgG1 (abcam, CT6).

### Image Analysis

Images were processed with the open source image analysis software, JEX [26] and associated JEX plugins for CTC analysis [28]. In JEX, individual 10x images were stitched into large images of the entire device or slide chamber. Average background fluorescence was subtracted from each channel within each large image prior to subsequent analysis. Cells were identified and masked based on user-defined Hoechst staining thresholds, then further evaluated for average cellular staining intensity for all other fluorescent antibodies. Data was output and analyzed in R, Microsoft Excel, and GraphPad Prism.

In flow cytometry, staining intensity is compared between different cells by graphing each cell as an individual data point on a scatter plot. When graphed in this way, cells distribute into higher and lower density regions, where cells with similar staining characteristics cluster together [29]. These cell clusters are surrounded by low density borders, which divide clusters, and allow analysts to manually define boundaries with gates. Manual gating is widely accepted in flow cytometry and allows more complex interrogation of staining characteristics on population subsets. In this report, thresholding for positive and negative antibody staining in fluorescence microscopy was based on clustering of cell populations within a representative patient sample. These thresholds were then applied uniformly across all patient samples treated with similar experimental conditions.

### Statistical Analysis

For flow cytometry, mean fluorescence intensity (MFI) values of populations of interest were extracted from FlowJo software and statistically analyzed in Excel. Individual data points collected during flow cytometry are contained in FCS files. Fluorescence microscopy data were extracted with JEX, tabulated with R, and imported and statistically analyzed in Excel. Individual data points collected from JEX were tabulated by R into “.arff” files. T-tests were used to evaluate the significance of data from both flow cytometric and fluorescence microscopy analyses. T-test parameters are described in greater detail in associated figure legends and S2 Table.

## Results

In this study, CTCs were isolated from patients with NSCLC using ESP technology [1, 2]. Prior to antibody-based capture, ficoll gradients were used to isolate PBMCs from more dense populations of granulocytes and red blood cells (RBCs). In contrast to healthy individuals, immune cells in patients with cancer become activated, thus causing populations such as granulocytes to change density and co-purify with the PBMC fraction in the buffy coat [15]. Considering previous reports that cytokeratin stains neutrophils [16], we evaluated the potential for these cells to be misidentified as CTCs.

**Fig 1:**
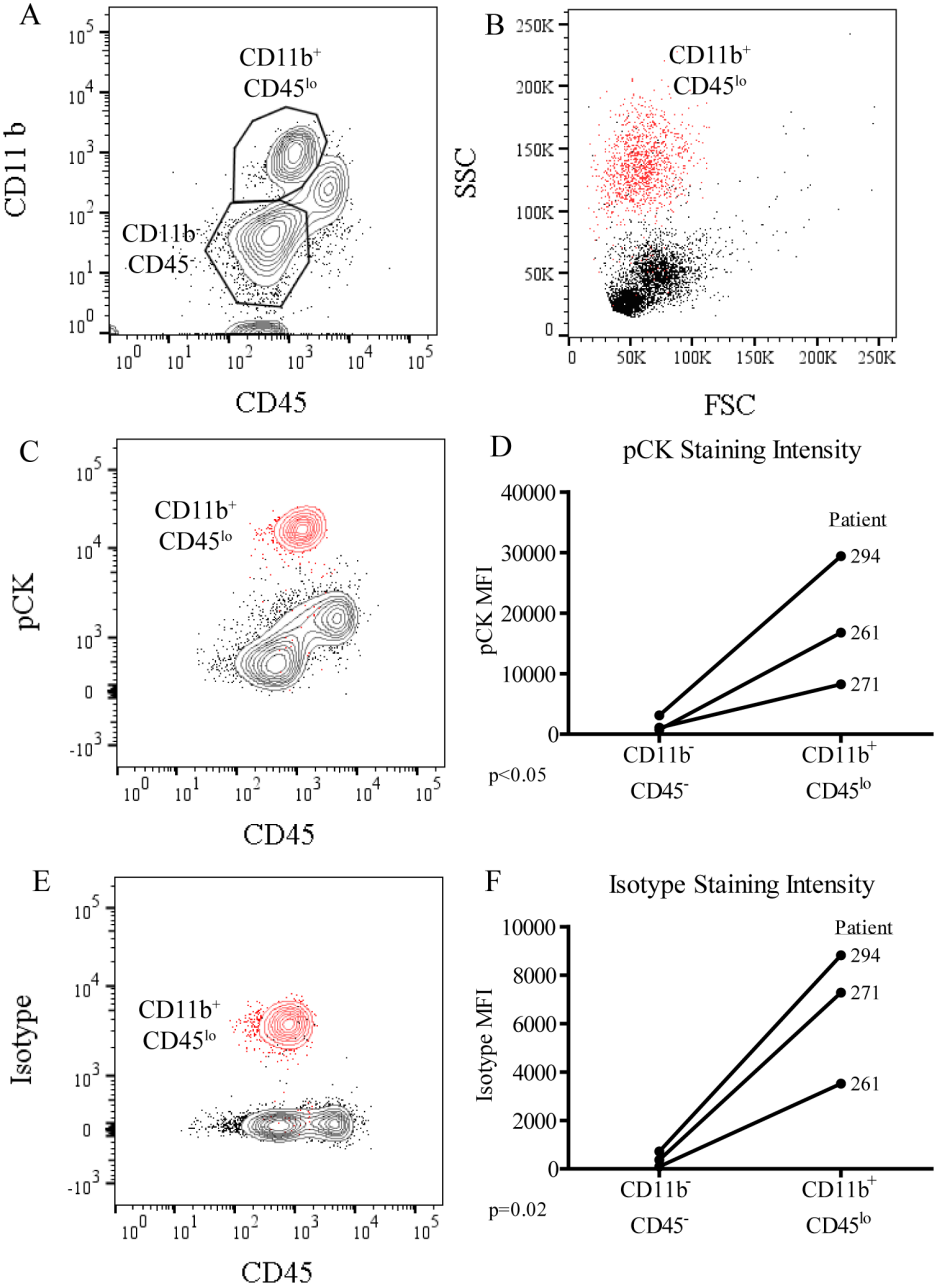
Flow cytometry reveals many CD11b^+^CD45^-^ cells are also pCK^+^. PBMCs isolated from patients with NSCLC and depleted of CD45^+^ cells were stained for flow cytometric evaluation. (A) Cells were gated first on live cell populations, then on cells which were either CD11b^+^CD45^lo^ or CD11b^-^CD45^-^. (B) Subsequent plots displayed previously gated CD11b^+^CD45^lo^ cells (red) over all other live cells (black) on X and Y axes of FSC vs. SSC, (C) CD45 vs. pCK, or (E) CD45 vs. the isotype control for pCK, IgG1. (D) This staining and gating strategy was performed over three different patient samples to evaluate staining intensity, or mean fluorescence intensity (MFI), of pCK or (F) the isotype control. Significance was evaluated by one-tailed, paired T-tests with significance considered at p<0.05 (*).

The presence of potential CTC false-positives was evaluated by flow cytometry prior to CTC isolation. Buffy coats from NSCLC patient samples were first depleted of the majority of CD45^+^ cells, then stained for CD45 and pCK in addition to CD11b, an antigen widely expressed by myeloid derivatives such as MDSCs and neutrophils [12]. By plotting all live cells on CD45 and CD11b axes (Fig 1A), distinct populations of CD11b^+^CD45^lo^ and CD11b^-^CD45^-^ cells were observed. When compared to all live cells, the population of CD11b^+^CD45^lo^ cells localized to the forward scatter (FSC) and side scatter (SSC) region characteristic of granulocytes such as neutrophils (Fig 1B). Further analysis revealed that the pCK staining intensity was greatest on this population of CD11b^+^CD45^lo^ cells when compared to all live cells (Fig 1C). CD11b^+^CD45^lo^ cells stained between 8 and 22 times greater for pCK than did CD11b^-^CD45^-^ cells (Fig 1D), with an average increase of 13-fold over three different patients. The strong pCK staining of these CD11b^+^CD45^lo^ cells demonstrates their potential to be misidentified as CTCs.

The FSC/SSC distribution of these CD11b^+^CD45^lo^ cells suggested they were of size and granularity that is characteristic of cells of granulocytic origin, cells which also tend to display elevated non-specific staining patterns, particularly after intracellular staining protocols. Therefore, the specificity of the intracellular pCK staining was assessed by staining with the isotype control, IgG1. Non-specific staining due to the isotype alone was significantly elevated in the CD11b^+^CD45^lo^ cells compared to the CD11b^-^CD45^-^ cells (Fig 1E and 1F), ranging from 12 to 38-fold higher, with an average factor of 23-fold. Differences between these two populations, therefore, reflected differences in staining specificity, rather than antigen expression; and that despite elevated staining for pCK, these CD11b^+^CD45^lo^ cells should not be considered CTCs.

**Fig 2:**
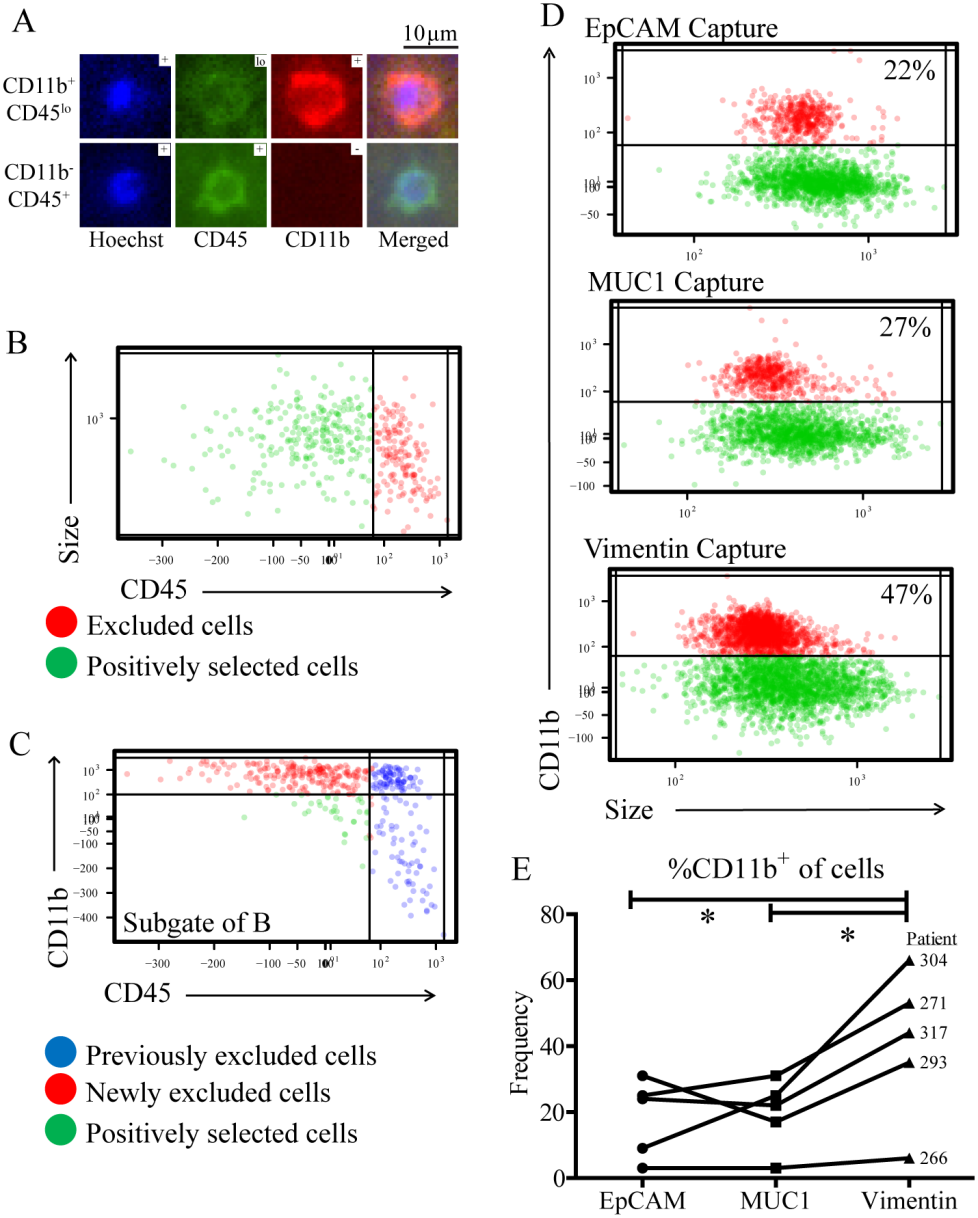
CD11b^+^ cells are present after antibody-based CTC capture. Cells were isolated from NSCLC patient samples using an antibody against MUC1. (A) Cells were then stained and imaged with a fluorescence microscope and crops of representative 10x images were given as examples of CD11b^+^CD45^lo^ or CD11b^-^CD45^+^ cells. (B) Cells were identified and analyzed in JEX, then graphed in R with each cell represented as one point. Cells were displayed on an X-axis of mean fluorescence intensity (MFI) of CD45 staining, and a Y-axis of cell size. CD45^+^ cells (red points) were excluded by manual gating on clustered populations. (C) Subsequent gating in R displayed previously excluded cells (blue) with newly excluded cells (red) and positively selected cells (green) on X- and Y-axes of CD45 and CD11b MFI, respectively. Gating in this plot was based on exclusion of the population of cells which emerged from additional CD11b staining. (D) Cells were isolated from one NSCLC patient sample in three parallel experiments using either an antibody against EpCAM, MUC1, or Vim. The average frequency of CD11b^+^ cells isolated from different antibodies was 22, 27 and 47%, respectively. (E) Results from five different patients were compared using one-tailed, paired T-tests with significance considered at p<0.05 (*).

If these CD11b^+^CD45^lo^ cells were to contaminate CTC isolations, they could be misidentified as CTCs based on staining for pCK and CD45 alone. To evaluate whether or not any of these CD11b^+^CD45^lo^ cells were present after CTC isolation, CTCs were captured with ESP technology. As NSCLC patient samples may require capture by EMT antibodies, CTCs were captured with antibodies against MUC1, and antigen which is expressed by CTCs of both epithelial and mesenchymal phenotype [30]. After capture, the final CTC-enriched fraction was stained and imaged to reveal distinct populations of both CD11b^+^CD45^lo^ cells, and CD11b^-^CD45^+^ cells (Fig 2A). Images of cells were processed with JEX image analysis software to identify cells and quantify antibody staining. Information from JEX was then imported into R where sequential gating strategies allowed exclusion and selection of individual cells based on staining characteristics, similar to data analysis techniques used in flow cytometry [29]. By first plotting cells based on CD45 expression and size characteristics (Fig 2B), gates were used to exclude cells which expressed CD45. Subsequent plotting revealed a distinct population of cells which could be further excluded based on the expression of CD11b (Fig 2C). These plots demonstrate the presence of distinct populations of cells which strongly expressed either CD45 or CD11b within the cell fraction obtained after CTC capture.

As EMT antigens are expressed by many myeloid subsets, using EMT antibodies such as MUC1 to capture CTCs could lead to elevated CD11b contamination. To evaluate the impact of EMT capture on CD11b contamination, EMT antibodies targeting either MUC1 or Vimentin (Vim), were compared to traditional EpCAM-based capture. The effects of different capture antibodies, therefore, were compared within individual patient samples, using antibodies against either EpCAM, MUC1, or Vim (Fig 2D). Gating on CD11b^+^ cells revealed that 22, 27, and 47% of the cells captured by these antibodies, respectively, were CD11b^+^ cells. This experiment was repeated over samples from five different patients with NSCLC (Fig 2E), where between 3 and 66% of captured cells were CD11b^+^, depending on the patient and capture antibody used. CD11b^+^ cells comprised, on average, 41% of the population captured with Vim, as opposed to 18 and 20% from EpCAM and MUC1, respectively. Overall, Vim captured significantly more CD11b^+^ cells than either MUC1 or EpCAM, while no significant difference was observed between the latter two. Therefore, despite MUC1 targeting CTCs of both EMT and epithelial phenotypes, MUC1 did not capture a significantly different frequency of CD11b^+^ cells than traditional capture with EpCAM.

**Fig 3:**
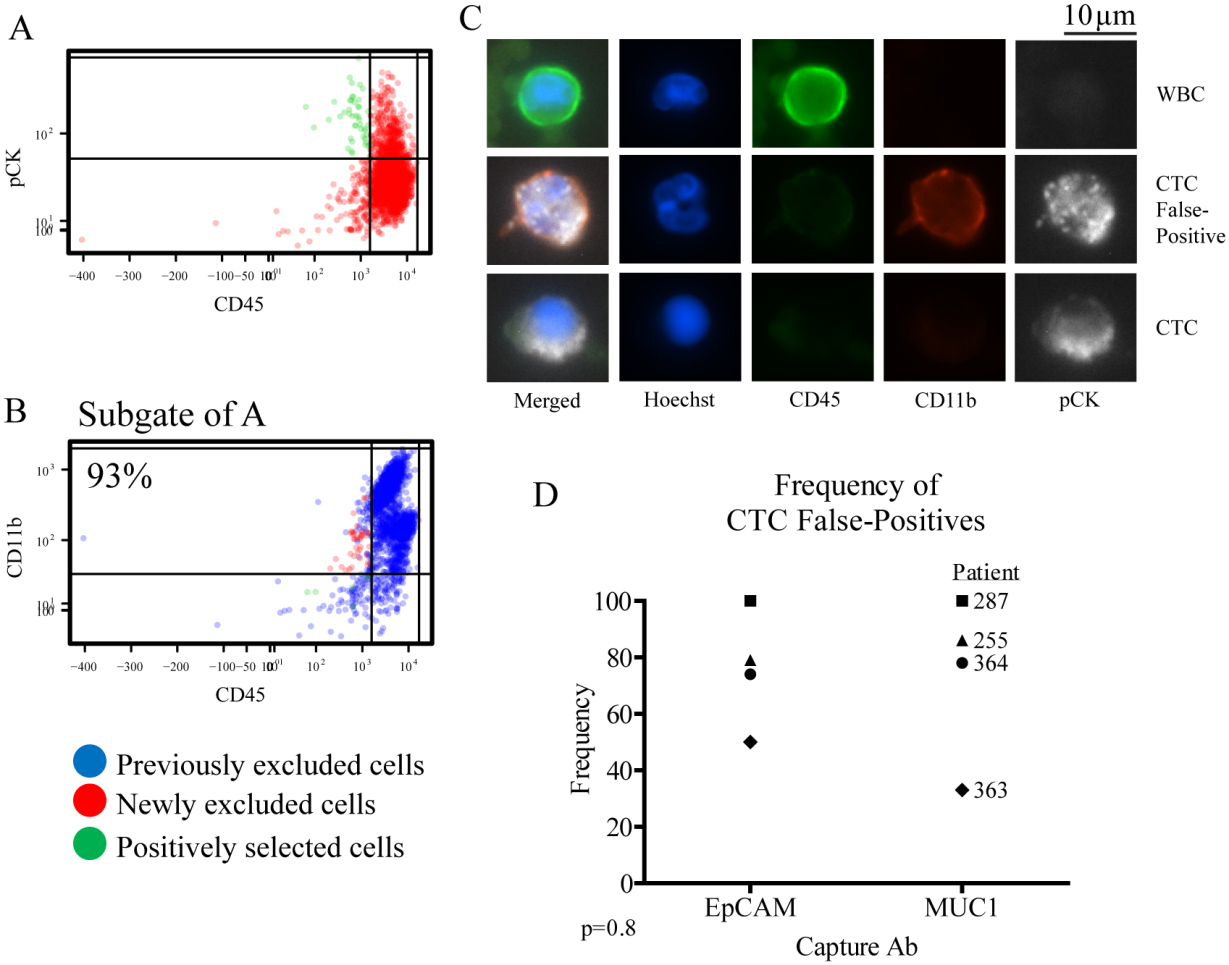
CD11b staining increases specificity of CTC identification. CTCs were captured from CD45 depleted buffy coats with MUC1 or EpCAM labelled PMPs, then stained and identified in JEX and R based on CD45 and pCK staining. (A) pCK^-^CD45^+^ cells were excluded in the first gate (red dots), while pCK^+^CD45^-^ cells were positively selected (green dots) as traditionally identified CTCs. (B) Subgated plots enabled further exclusion of CD11b^+^ cells (red dots), from CD11b^-^ cells positively selected as CTCs (green dots) displayed over previously excluded cells (blue dots). Red dots in this subgate represent false-positives. (C) Example 40x images were shown for a WBC (CD11b^-^CD45^+^pCK^-^), a CTC false-positive (CD11b^+^CD45^lo^pCK^+^), and a true CTC (CD11b^-^CD45^-^pCK^+^). (D) Data from multiple samples were graphed as individual data points to demonstrate the frequency of false-positives using either EpCAM or MUC1 capture antibodies. Results from four different patients were compared using a two-tailed, paired T-test.

To determine whether or not these CD11b^+^ cells could be misidentified as CTCs, cells were captured with either MUC1 or EpCAM, then classified as CTCs based on staining for pCK and CD45 alone (Fig 3A). Subsequent gating revealed a significant population of cells previously identified as CTCs were actually CD11b^+^ cells (Fig 3B). CD11b^+^ cells were falsely identified as CTCs after capture by either MUC1 or EpCAM, in 33–100% of those identified as CTCs, with an average of 76 and 74% from either EpCAM or MUC1 capture antibody, respectively, over four different samples from patients with NSCLC (Fig 3D). There was no significant difference between EpCAM and MUC1 for the frequency of CTC false-positives (Fig 3D). These results demonstrate the potential for CD11b^+^ cells to be captured by either MUC1 or EpCAM and then misidentified as CTCs based on pCK and CD45 staining characteristics alone.

**Fig 4:**
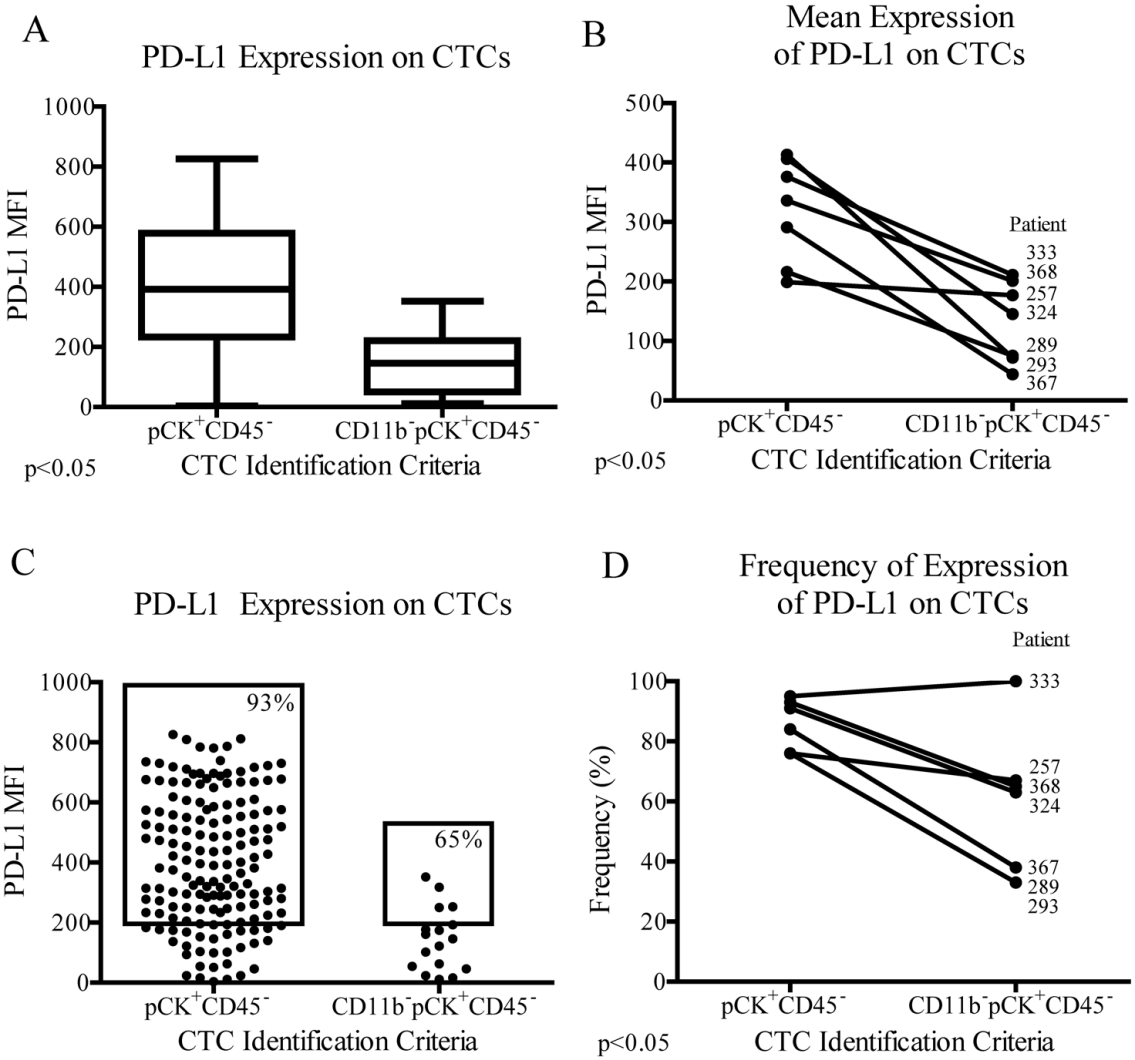
High specificity in CTC identification affects PD-L1 evaluation. (A) CTCs captured with MUC1 and identified from patient 324 as either pCK^+^CD45^-^ or CD11b^-^pCK^+^CD45^-^ were graphed as individual data points and compared for average PD-L1 MFI and (C) frequency of PD-L1^+^ CTCs. (B) Data from seven different patient samples were represented as seven single data points and compared by CTC identification criteria, either with or without CD11b exclusion criteria, for average PD-L1 expression and (D) frequency of PD-L1 expression on CTCs. Results were compared using two-tailed T-tests; unpaired for each individual sample, and paired for overall comparisons.

As MUC1 and EpCAM captured similar frequencies of CD11b^+^ cells and CTC false-positives, only MUC1 was used to evaluate the impact of CTC misidentification on PD-L1 evaluation. Of cells captured with MUC1 and identified as CTCs by standard criteria (pCK+CD45-), PD-L1 expression level was significantly higher in patient 324 when CTC false-positives were excluded with CD11b (Fig 4A). This trend varied slightly over multiple patient samples, where CD11b exclusion had varying degrees of impact on the observed CTC PD-L1 expression level (Fig 4B). Variability in the effect of CD11b exclusion was also seen when CTCs were quantified for the frequency of PD-L1 expression (Fig 4C and 4D). Depending on the CTC identification criteria used, patients might be incorrectly classified as having either high or low PD-L1 CTC expression.

## Discussion

There is a critical need for predictive and pharmacodynamic biomarkers in aggressive diseases such as NSCLC. Despite the great potential of CTC analysis to help address this need, there has been limited success. Success in this area will require robust and specific CTC identification to avoid skewing from non-CTCs during biomarker evaluation. For example, in the case of NSCLC, checkpoint inhibitors that target immunomodulatory proteins such as PD-L1 complicate CTC-based screening as PD-L1 and L2 can be expressed on both tumor cells and other blood cells. As shown recently, CTC identification using CD45 and EpCAM can generate 21% false-positives [31]. If any of those falsely identified cells also highly express PD-L1, then assessment of PD-L1 for that patient could be dramatically skewed. A similar problem exists for standard enumeration of CTCs, based solely on staining for CD45 and pCK. Up to this point; standard enumeration has failed to consider cells in the early stages of hematopoietic differentiation, those arising prior to the expression of CD45, which also proliferate in patients with cancer [12, 13]. Even some mature myeloid derivatives like neutrophils, which express low levels of CD45, also increase in frequency as cancer progresses [15], raising the potential to mistake them for CTCs. Previous studies have shown that purified neutrophil granulocytes can stain positively for cytokeratin [16], thus emphasizing their potential to be falsely identified as CTCs were they to contaminate CTC isolations.

Here, we identified a CD11b^+^ CD45^lo^ population that stains far brighter for pCK than the fraction of CD11b^-^CD45^-^ cells which would theoretically include CTCs. These CD11b^+^ cells are likely of myeloid origin: either immature myeloid or neutrophil granulocytes, since they tend to be large and granular. But the pCK^+^CD45^-^ staining profile would be similar to that traditionally attributed to CTCs, especially under visual interpretation of fluorescence microscopy images. Therefore, CD11b^+^CD45^lo^ cells could be falsely identified as CTCs were any of them to contaminate the product of CTC isolation. Isotype staining was also much brighter on this CD11b^+^ subset compared to the CD11b^-^ subset, therefore, pCK staining was largely non-specific, suggesting this population of cells does not actually express pCK, and does not actually represent a subset of CTCs. CTC identification with pCK may be effective, but due to the elevated non-specific pCK staining on CD11b^+^ cells, additional exclusion criteria is essential for accurate enumeration.

The cytokeratin clone used here, C-11, has been used in many publications to identify CTCs, and is therefore relevant to many researchers [32–36]. The C-11 clone has specificity for cytokeratins 4, 5, 6, 8, 10, 13 and 18. The cytokeratin cocktail used by CellSearch, in contrast, only recognizes cytokeratins 8, 18 and 19 and may be more specific for epithelial cells than the former. The non-specific staining observed here, however, is associated with an intracellular staining artifact of CD11b^+^ cells which is independent of the specificity of the antibody. The non-specific nature of this staining suggests any intracellular cytokeratin antibody, including that used by CellSearch, may contribute to misidentification of CD45^lo^CD11b^+^ cells as CTCs.

The observation that different CTC targeted antibodies captured different amounts of CD11b^+^ cells, demonstrates how EMT targeted antibodies could further amplify this dilemma. Diversity amongst patients in how they are affected by different capture antibodies may also be attributable to variation in the frequency of neutrophils [15] and MDSCs [13] with disease progression. The significant elevation in CD11b^+^ cells captured by Vimentin compared to either EpCAM or MUC1, and the fact that neutrophils express Vimentin, but not EpCAM or MUC1, suggests these additional CD11b^+^ cells are likely neutrophils.

Importantly, accurate identification of CTCs extends across nearly all CTC platforms, whether antibody based capture, size exclusion or other negative selection modality [37–39]. In this study, cells captured with EpCAM and MUC1 contained approximately 20% CD11b^+^ cells, despite neither EpCAM nor MUC1 being expressed by neutrophils. These CD11b^+^ cells could be other myeloid derivatives, as CD11b is expressed during the early stages of myeloid differentiation. Myeloid cells also express Fragment crystallizable (Fc) receptors, which are commonly associated with non-specific binding artifacts, even after the use of blocking proteins such as BSA. Non-specific binding could also be mediated by the adhesive properties of myeloid cells, and their ability to bind to molecules such as fibronectin [40]. Fibronectin bears significant sequence similarity to streptavidin [41], a molecule which is frequently used in immunological assays. In this study, antibody labelled streptavidin PMPs were used to capture CTCs, potentially also enriching for fibronectin-binding myeloid derivatives.

These CD11b^+^ cells formed clustered distributions on R scatter plots of JEX intensity data, indicating that these cells are a defined population. Fluorescent staining inherently manifests itself as a gradient of fluorescence intensity, not as a binary division between positive and negative staining. Therefore, distinguishing between positive and negative staining requires the ability to set thresholds between populations, or clusters of cells with distinct intensity ranges. Clustering is used to identify distinct populations of cells in flow cytometry [29]; similarly, here we identified a population of CD11b^+^CD45^lo^ cells with unique characteristics from that which highly expresses CD45. The CD11b^+^CD45^lo^ staining is characteristic of cells of myeloid origin, distinct from other hematopoietic cells, and not representative of epithelial cells such as CTCs.

In this report, we demonstrate that the pCK/CD45 staining similarities between CTCs and myeloid derivatives prevents a clear differentiation without additional staining for CD11b. Myeloid-specific markers, such as CD11b, add separation to overlapping populations, reducing potential CTC false-positives. Without this additional staining parameter, only two populations of cells can be distinguished, where myeloid cells would be incorrectly grouped together with CTCs. Cells which may seem pCK^+^CD45^-^ in contrast to their counterpart, pCK^-^CD45^+^ cells, may actually be CD11b^+^, and not true CTCs. Only by adding a third parameter, such as CD11b, can the population of pCK^+^CD45^-^ cells be divided into true CTCs and false-positive myeloid derivatives.

In our study, these myeloid derivatives were misidentified as CTCs to a similar extent after either EpCAM or MUC1 based capture, at 76 and 74%, respectively. By excluding CD11b^+^ cells, the observed CTC PD-L1 expression level increased to varying degrees in different patients, supporting the idea that inter- patient variability in disease state may affect the frequency of interfering WBC populations. CTC identification with markers other than pCK, such as MUC1, may be more specific in contrast to the non-specific nature of intracellular pCK staining. Extracellular staining with MUC1to identify CTCs was evaluated in S1 fig and suggests fewer CTC false-positives with this approach. These false-positives had a similar impact on the observed CTC PD-L1 expression level, with variability in magnitude and significance between different patient samples. Thus, the extent of CTC misidentification, or disease severity, accordingly, could have variable levels of impact on the resulting evaluation of the biomarker.

After excluding CD11b^+^ events from CTC identification, a dynamic range is observed within both average and frequency of PD-L1 expression on CTCs, demonstrating the potential to detect PD-L1 variation in the context of prospective clinical trials. Dynamic range allows stratification of the patient population in order to correlate response to treatment, where patients are grouped into positive or negative predictive categories. CTC PD-L1 quantification can be skewed by CTC false-positives, however, significantly changing the interpretation of the PD-L1 status. The amount of CD11b false-positives varies between patients, as well as the effect those false-positives have on the observed amount of CTC PD-L1. Therefore, to obtain clinically useful data, we must quantify PD-L1 on CTCs specifically, and exclude interfering populations. Eliminating these false-positives increases the accuracy of the PD-L1 evaluation, and ultimately the clinical relevance of the assay.

CD11b was chosen over other markers as it is highly expressed at an early stage of myeloid development. Other markers, such as CD15, CD16, etc., are expressed by many myeloid cells, but typically at much lower levels than CD11b. Relative expression values for these markers were extracted from biogps.org [42] and compiled in S3 Table. Biogps.org also suggests that while CD11b is expressed to some degree on lung cells, it is more strongly expressed by myeloid-derived cells. As these databases pool information derived from analysis of whole tissues, it is feasible that neutrophil infiltration in the lungs could contribute in part to elevated CD11b levels in this tissue. Positive and negative expression cutoffs in our analysis were based on interpretation of clustering populations, those displaying large differences in expression level. By this method, a minimal level of CD11b expression on CTCs would be considered negative in contrast to that of strongly expressing myeloid populations.

There is a risk of excluding putative CTCs, thus lowering sensitivity, with this approach. For example, tumor-associated neutrophils (TANs) could be bound to CTCs in the circulation [43], excluding these events from analysis. In our microscopy analysis, however, cells were identified with automated software analysis program, JEX. This software first identifies cells based on thresholds for nuclear stain, where cells in close proximity are separated based on individual nuclei. Each identified cell is further verified by manual inspection within the associated R analysis application. Using CD11b to eliminate CTC false-positives would therefore not exclude any additional true CTCs were they bound to TANs.

In conclusion, our data demonstrates how myeloid derivatives which are misidentified as CTCs will skew biomarker evaluation. Staining for CD11b excludes many interfering cells, and improves the specificity of CTC identification. By refining this population, CTC biomarker analysis becomes more accurate, with greater capability to predict response to treatment. Automated cell capture and image analysis technologies, ESP and JEX, provide a platform for sensitive and objective CTC evaluation, characteristics which are essential for assay validation as companion diagnostics. Improvements in the reliability of CTC analysis will allow us to harness the true diagnostic potential of a liquid biopsy to guide clinical decision making.

## Supporting Information

S1 Fig CTC false-positives are captured and identified with MUC1. Samples from NSCLC patients were processed with ESP technology as described above. (A) CTCs were captured with MUC1 and identified based on positive staining for MUC1 and negative staining for CD45 (green dots). (B) The subgate of A reveals 10% of those previously identified as CTCs were newly excluded by additional staining for CD11b (red dots). (C) The frequency of CTC false-positives ranged from 10–20%, with an average of 13%. (D) CTCs Identified with MUC1 and CD45 alone in patient 289 had a significantly higher average PD-L1 MFI than those identified with the additional CD11b exclusion criterion. (E) CD11b exclusion had varying degrees of impact on the observed PD-L1 MFI of CTCs, with up to a 3-fold significant decrease in some patients, and no significant change in others.
(TIF)

S1 Table Patient disease characterization and treatment response at time of blood draw. Details of disease and treatment response were compiled for each patient. Disease type and stage were determined clinically by histological examination. Mutational or PD-L1 expression status was based on analysis of tumor biopsies, if performed. Treatment and observed response were listed for the time of blood draw for samples analyzed and presented in the corresponding figures above.
(XLSX)

S2 Table Datasets used in graphs and statistical analyses. Data was compiled for figures and statistical references from the associated figures. Data was obtained from interpretation of fluorescence microscopy images and flow cytometry files using either JEX, R and Excel, or FloJo software. JEX and FCS source files are available upon request.
(XLSX)

S3 Table Compilation of relative expression of different myeloid markers. Values posted on biogps.org were compiled for various markers associated with identification of myeloid subsets. These values correspond to mRNA expression levels, which may not always correlate absolutely with protein expression levels, and are meant to provide a rough comparison of relative expression.
(XLSX)
