# Supplementary figures and images for: High Specificity in Circulating Tumor Cell Identification Is Required for Accurate Evaluation of Programmed Death-Ligand 1
Source: PLoS One. 2016 Jul 26;11(7):e0159397. doi: 10.1371/journal.pone.0159397 (PMC4961410; doi:10.1371/journal.pone.0159397)

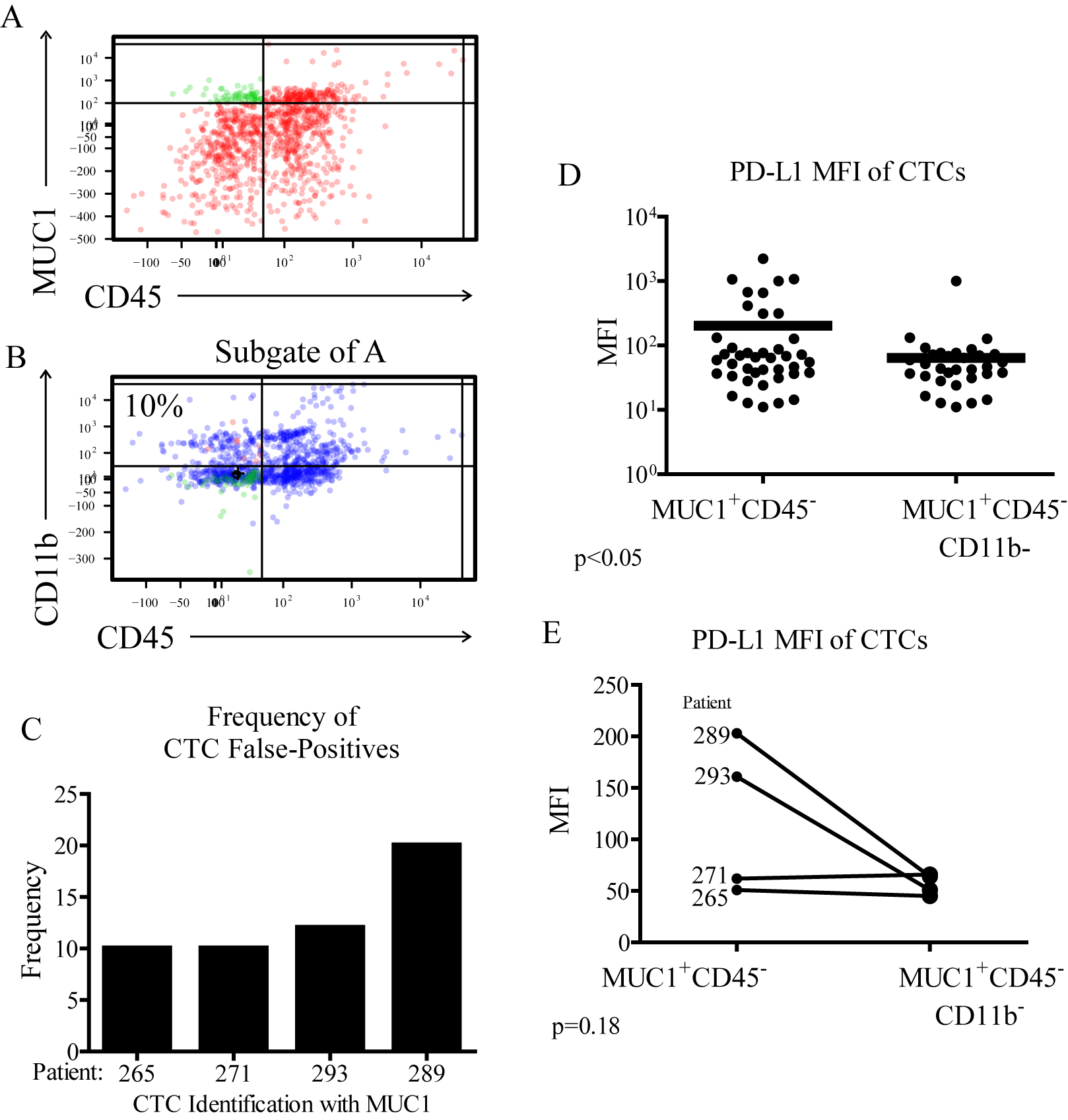

Supplement: S1 Fig — Samples from NSCLC patients were processed with ESP technology as described above. (A) CTCs were captured with MUC1 and identified based on positive staining for MUC1 and negative staining for CD45 (green dots). (B) The subgate of A reveals 10% of those previously identified as CTCs were newly excluded by additional staining for CD11b (red dots). (C) The frequency of CTC false-positives ranged from 10–20%, with an average of 13%. (D) CTCs Identified with MUC1 and CD45 alone in patient 289 had a significantly higher average PD-L1 MFI than those identified with the additional CD11b exclusion criterion. (E) CD11b exclusion had varying degrees of impact on the observed PD-L1 MFI of CTCs, with up to a 3-fold significant decrease in some patients, and no significant change in others. (TIF) [file pone.0159397.s001.tif]
